# Knowledge and Farmers’ Adoption of Green Production Technologies: An Empirical Study on IPM Adoption Intention in Major Indica-Rice-Producing Areas in the Anhui Province of China

**DOI:** 10.3390/ijerph192114292

**Published:** 2022-11-01

**Authors:** Xiaolong Sun, Jing Lyu, Candi Ge

**Affiliations:** 1 Institute of Agricultural Economics and Development, Jiangsu Academy of Agricultural Sciences, Nanjing 210014, China; 2 Jiangsu Agricultural Science and Technology Innovation Decision Consulting Research Base, Nanjing 210014, China; 3 Beijing National Accounting Institute, Beijing 101318, China

**Keywords:** IPM technology, production knowledge, pest control, rice production, pest management knowledge, nutrient management knowledge, agro-environment knowledge, cultivation technology knowledge

## Abstract

As a comprehensive technology with social, economic, and ecological benefits, integrated pest management (IPM) is crucial in fundamentally alleviating the environmental pollution caused by traditional high-input agriculture. Based on the random-sampled data of 981 farmer households in major Indica-rice-producing areas in Anhui Province, this study analyzes the impact of agricultural production knowledge on farmers’ willingness to adopt IPM technology through logit models, considering integrated knowledge and categorized knowledge. The results indicate that integrated agricultural production knowledge significantly increases farmers’ willingness to adopt IPM technology. However, pest-management knowledge was the only one out of four specific disciplines that significantly individually affect farmers’ adoption intention. The more knowledge farmers acquire about pest management, the higher intention they have to adopt IPM. Some demographic and household characteristics also significantly influence their willingness. Based on these results, we suggest that increasing farmers’ agricultural production knowledge, especially knowledge about pest management, is essential in promoting IPM technology. Besides this, IPM technology should be promoted purposely and consciously, combined with farmers’ individual and family characteristics.

## 1. Introduction

In a situation of an ever-increasing world population and more intensive agriculture, food security is of constant concern globally. From the angle of production, it is of great significance to avoid crop loss from pathogens, diseases, pests, and weeds. For decades, farmers utilized chemical pesticides/herbicides for disease, pest, and weed control [1]. However, for multiple reasons, e.g., a lack of pest-management knowledge [2,3], misleading information [2,3], the pursuit of high crop yield [4], and pest resistance [5], the misuse of pesticides/herbicides (including incorrect application, overuse, underuse, and use of restricted or even banned products), agricultural and environmental sustainability and human health have been heavily threatened, especially in developing countries [3]. Misuse of pesticides also causes pest resistance, the emergence of new pests, and the destruction of beneficial insects [5,6]. For agricultural sustainability, stakeholders, agricultural professionals, and policymakers have paid considerable efforts to alternative and more environment-friendly pest-control methods [7,8].

Integrated pest management (IPM), as a sustainable pest-management approach, has gained much attention. In general, IPM refers to a comprehensive science-based decision-making process that identifies pest-relative risks and coordinates multiple disciplines to prevent and control pest damages using the most economical means, at the same time relieving stresses on humans, property, resources, and the environment [9]. IPM, allowing farmers to manage pests in a cost-effective and environmentally-friendly way, involves biological control, physical control, agricultural control, and scientific ways of using pesticides [10,11]; thus meeting the requirements of sustainable development and being suitable to adopt under local conditions [12]. Applying IPM reduces pesticide use [7,13,14,15,16], saves input costs [16,17], increases crop yields [7,14,15,17], protects the environment [11,13], ensures the quality of agricultural produce [11], and brings extra economic benefits [11,13,14]. Because of multiple benefits of IPM, ways of boosting its application have attracted significant interest from various stakeholders.

Since farmers are decision-makers, it is important to identify their IPM-adopting behavior. According to previous literature, agricultural production factors, such as farm size [10,18,19,20,21], farming experience [10,19], number of farm-labor forces [10,20], and non-agricultural involvement of the farm household [10,21,22] significantly influence farmers’ IPM adoption. Farmers’ risk perception and environmental awareness both influence their adoption behavior. Risk-averse farmers are less likely to adopt IPM [22]. Meanwhile, if farmers care more about the environment, they are more likely to adopt IPM in agricultural production [22,23]. Market incentives and governmental supports are also important factors in farmers’ decision-making [23]. Besides this, the availability of loans [22], the availability of extension activities [19], and promotion strength [20] all enhance farmers’ IPM adoption.

Last but not least, as a compounded agricultural technique, IPM application requires appliers to be familiar with relevant knowledge. According to specific field conditions, IPM involves techniques in cultivation, soil management, mechanical control, biological control, chemical control, etc. [9,14,19]. To adopt IPM, farmers have to master skills in pest monitoring, crop science, biology, ecology, etc. [5]. Knowledge is crucial to farmers’ IPM adoption [2,7,19]. As IPM is knowledge-intensive [2,15], lack of knowledge might hinder farmers’ IPM adoption [24,25]. Though researchers include farmers’ knowledge when investigating the impact factors of farmers’ IPM adoption, they usually use farmers’ education levels as an indicator of farmers’ knowledge [10,18,22]. There are two limits for using farmers’ education level for their agricultural production knowledge. First, education level and the level of agricultural production knowledge may not be completely correlated. Second, even though it is hard to increase farmers’ education level, it is possible to increase farmers’ knowledge through training. Some other scholars include farmers’ training experiences in their IPM-adoption analyses and conclude that farmers who have training experiences, such as FFS, IPM technology training, and agricultural training, are more likely to adopt IPM approaches compared with untrained farmers [10,18,21,22]. However, using farmers’ training experience to represent their agricultural knowledge is not rigorous either.

There is limited research investigating how farmers’ knowledge influences their IPM adoption. Pouratashi and Iravani [26] assessed farmers’ IPM knowledge using four attributes including the negative aspects of pesticide use, awareness of pest-control alternatives, knowledge of beneficial insects, and the definition of IPM. However, they did not evaluate the impact of knowledge on IPM adoption. Including a self-reported question on a scale of five, Allahyari et al. [19] evaluated the effect of farmers’ knowledge on their IPM adoption from a more niche-targeting point of view, focusing on the techniques of IPM. Yaguana et al. [2] examined the long-term effect of farmers’ IPM knowledge on their IPM adoption over ten years and concluded that farmers’ IPM knowledge promoted their adoption even over a long period. Gautam et al. [7] evaluated the correlation between farmers’ knowledge and their adopted IPM practices. However, they focused solely on farmers’ knowledge of insect pests. Even though Liu et al. [23] asked farmers three questions to evaluate their knowledge of IPM technologies, the three questions were somewhat shallow, and may not imply farmers’ real perception of IPM technology. In this study, we test farmers through 45 questions concerning four disciplines of knowledge related to agricultural production and IPM to obtain scores that more precisely reflect their knowledge levels.

Rice (*Oryza sativa* L.) is one of the major staple grains in China, with a total yield achieving 211.86 million tons [27]. This high yield of rice was associated with a huge usage of chemical pesticides and fertilizers [3]. In 2019, commercial pesticide usage in China was 1.46 million tons [28], counting for approximately 12% of the world’s total [29]. The utilization rate of pesticides in China is about 39.8% for rice, maize, and wheat [28], causing severe environmental and health problems [30], not to mention the misuse and overuse of pesticides to ensure rice yield [3,5,30]. Misuse of pesticides severely threatens the sustainability of pest control, farmers’ health, and the environment [31], causing high input costs and pest resistance, as well as the elimination of the natural enemies of pests [30]. To ease the threats caused by pesticide misuse and overuse, the Chinese government has promoted IPM for decades. In the late 1970s, the Chinese government promoted crop IPM programs with a top-down extension approach. However, as the land policy changed to the household responsibility system in the 1980s, a huge number of smallholder farms became agricultural business entities. Thus, the top-down IPM approach was replaced by national IPM programs supported by international organizations. China participated in the Inter-Country IPM program for Rice from Food and Agriculture Organization (FAO) in 1988, introducing the farmer-led IPM tactics through the Farmer Field Schools (FFS) and the Training of Trainers program [5]. At present, the farmer-led IPM is still the major IPM approach in China, but with a low application rate [23].

Agricultural production in China relies on a large number of small-scale farmers, who are commonly less educated and focus mostly on crop yield. Though IPM might increase production yield [7], it is better known as an environment-benefiting agricultural technique. Thus, farmers may not have internal motivations to apply IPM and protect the environment. At present, environmentally-friendly production is a credence attribute that helps agricultural products gain higher values in the market. However, farmers might be insufficiently educated to obtain the credence value or to apply IPM and other environmentally friendly production techniques. The general purpose of this study is to determine the role of agricultural knowledge in farmers’ adoption intention of environmentally friendly agricultural production techniques—specifically, IPM technology. We contribute to the existing literature in the following aspects: 1. Instead of using farmers’ educational level or training experiences as an indicator of their knowledge, we gave farmers an exam and used the obtained scores as their knowledge indicator, which more reasonably and appropriately reflects farmers’ knowledge level; 2. When designing the questions for the exam paper, we considered the comprehensiveness and integrity of IPM technology. We include four disciplinary subparts of questions, i.e., pest management, nutrient management, agro-environment, and cultivation technology. Overall, in this study, we not only focus on the role of general knowledge but also the roles of disciplinary knowledge that might affect farmers’ IPM adoption. While a few previous studies have mentioned the importance of farmers’ knowledge to IPM adoption, few have considered farmers’ real agricultural production knowledge. Hence, this study makes a novel contribution to the literature by providing quantitative evidence for the effect of farmers’ agricultural production knowledge on their IPM adoption intention.

The analytical framework of this study is as follows. Section 2 summarizes the theoretical background. Section 3 describes the sampling procedure, questionnaire survey, and methods developed to examine farmers’ willingness to adopt IPM. In Section 4 we present descriptive statistics to investigate the relationship between farmers’ knowledge and their IPM adoption intention and the econometric regression results. Section 5 provides discussions, conclusions, and policy implications.

## 2. Conceptual Framework

Technology-adoption behavior is a key link in the process of agricultural technology innovation and diffusion. Previous research includes two categories according to the research objects. The first category focuses on the macro level of the overall situation of agricultural technology diffusion, including the mechanism, speed, and characteristics of diffusion, most of which adopt the “S” curve theory. The second category studies the factors impacting farmers’ technology adoption from the micro level, which is mainly based on a theory of farmers’ behavior. In this study, we focus on the impact of agricultural production knowledge on farmers’ willingness to adopt new technology, thus mainly referring to the theory of farmers’ behavior.

At present, the generally accepted theories about farmers’ behavior include the theory of rationality, the theory of irrationality, the theory of survival rationality, and the comprehensive theory of economic behavior [32]. However, many scholars believe that it is difficult to indicate farmers’ behavior based only on economic theories, as human behavior is a complex activity affected by many factors [33,34,35]. To better explain farmers’ behavior, some researchers propose to include psychological theories, such as including farmers’ psychological cognition as control variables in a theory of farmer’s behavior. Leeuwis and Ban [36] constructs a model based on the basic variables of individual behavior, trying to analyze farmers’ behavior from the perspective of cognition. In this study, we emphasize that “knowledge” is core to determining farmers’ behavior. Farmers’ knowledge of a technology should be identified or changed prior to a change in their adoption behavior of that technology [36]. Similar to evolutionary economics, which focuses on individuals’ heterogeneity, we emphasize the incompleteness of an individual’s knowledge, and believe that individuals always behave according to the limited knowledge they have at that specific time. In reality, farmers’ willingness to adopt new technology could differ because of the individual heterogeneity, which might be caused by different knowledge levels. Therefore, farmers’ understanding and levels of knowledge of new technologies should be enhanced to change their decision-making behavior.

From the perspective of economics, farmers’ willingness to adopt a certain technology depends on the comparative results between the marginal benefit and marginal cost of the technology. Farmers have to understand relevant information about the technology to a certain degree, as well as measuring the risks, benefits, and costs of adopting the technology (the cost of a technology mainly includes the expenses needed to adopt specific technology, and the opportunity cost of giving up the original technology in adopting the new one). The final adoption decision depends on farmers’ knowledge or experience of agricultural production. When farmers have weak agricultural production knowledge, the uncertainty (risk) of mastering new technology and potential benefits would be enlarged. Thus, they are reluctant to adopt new technology. In this study, we propose a research hypothesis as H_0_: the level of farmers’ agricultural production knowledge has a positive effect on their willingness to adopt new technologies. The more farmers know about agricultural production, the more willing they are to adopt new technologies.

## 3. Materials and Methods

### 3.1. Data and Research Design

#### 3.1.1. Description of the Study Region

**Figure 1:**
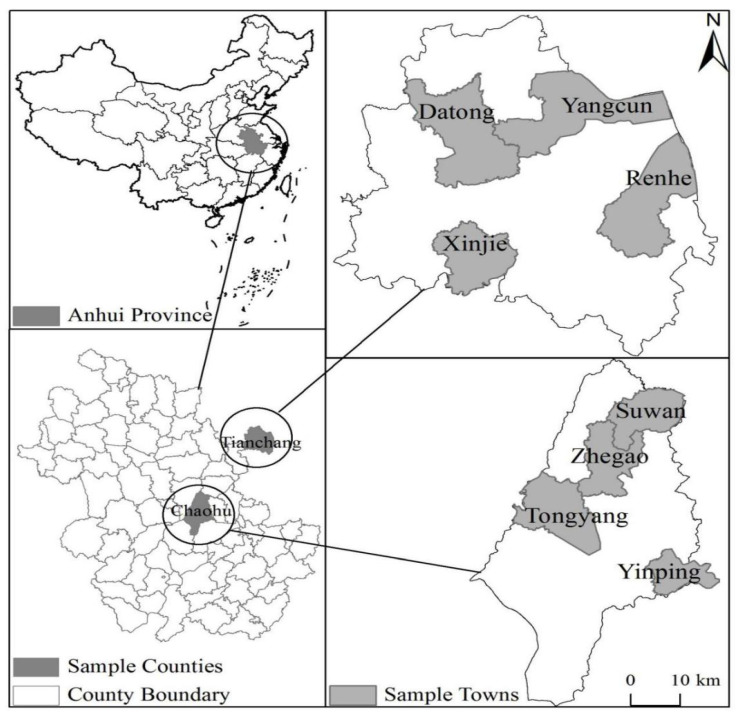
Location of the study regions.

The data were collected through a questionnaire focusing on rice farmers in the Anhui province of China, before the rice harvest season. Figure 1 represents the location of the surveyed area. The questionnaire mainly collected basic information about farmers’ households, technology input in rice production, rice yield, etc. Our respondents were household decision makers in agricultural production. To randomly select the samples, we first ranked all counties in Anhui using the rice-planting area per capita as an index, then randomly selected two counties, Tianchang and Chaohu. Tianchang and Chaohu are important grain-production bases, located in the east and the middle of Anhui Province, between the Yangtze River and the Huaihe River. The two counties are located in a hilly area aggregating hills, plains, and other terrains, with the altitude generally between 100 and 300 m. The research region is located in the transition zone from the north subtropical zone to the warm temperate zone, with four distinct seasons. Because of the abundant sunlight and rainfall, this area is suitable for the growth of Indica rice, considering the strong-light-resisting, heat-resisting, and moisture-proof characteristics of Indica rice. Thus, farmers in this area commonly grow Indica rice. Second, we randomly selected seven administrative villages from each of the four randomly selected towns out of each county. Finally, we randomly selected approximately 18 to 25 rice farmers in each village. The survey covered two counties, eight towns, 56 administrative villages, and 1171 respondents. Discarding the ones with missing values or mistakes, our sample includes 981 respondent data.

#### 3.1.2. Survey Design and Data Collection

**Figure 2:**
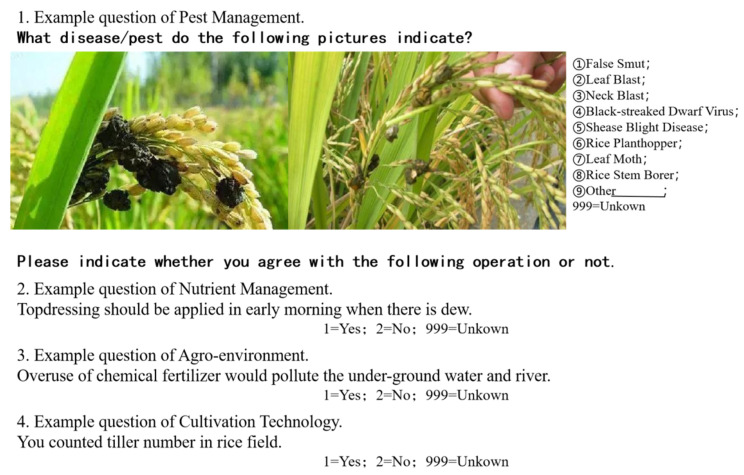
Sample questions from the four disciplines.

To assess farmers’ knowledge of agricultural production, we invited experts from the School of Agriculture, the School of Plant Protection and the School of Resource and Environment Protection of China Agricultural University to jointly set up a comprehensive test containing 45 questions (Please refer to Table S1 for a complete list of the test. Table S1 contains 47 questions. However, the last two questions were not considered a part of the test) on agricultural (medium Indica rice) production knowledge. The questions mainly include four disciplines: 27 questions on pest management, 6 questions on nutrient management, 4 questions on the agro-environment, and 8 questions on cultivation technology. Figure 2 summarizes a sample question from each discipline. For each discipline, all questions add up to a full score of 100 points, while the full score of the test is 400 points.

Table 1 summarizes the full scores and respondents’ average scores of the integrated test and four disciplines. From Table 1, we identify that our respondents commonly have a low level of agricultural production knowledge, with an average score of 161. Compared to other disciplines, farmers are more acquainted with the cultivation technology, followed by pest management and nutrient management. Farmers gained the lowest score in agro-environment, indicating that ecological environment protection is heavily neglected in agricultural production.

### 3.2. Methods

#### 3.2.1. Theoretical Model

*P_i_* stands for the probability of the *i*th respondent adopting IPM technologies, presented in Equation (1).

(1) $$P_{i}=\frac{1}{1+e^{-\left(\beta_{1}+\beta_{2}x_{i}\right)}}\;,$$

For more convenient analyses, we rewrite Equation (1) as:

(2) $$P_{i}=\frac{1}{1+e^{-z_{i}}},$$

The probability of the *i*th respondent not being willing to adopt IPM technologies is then:

(3) $$1-P_{i}=\frac{1}{1+e^{z_{i}}},$$

The odds ratio, calculated as the ratio between *P_i_* and (1 − *P_i_*), is

(4) $$\frac{P_{i}}{1-P_{i}}=e^{z_{i}}\;,$$

Taking the logarithm of the odds ratio, we obtain

(5) $$L_{i}=\ln\left(\frac{P_{i}}{1-P_{i}}\right)=z_{i}=\beta_{1}+\beta_{2}x_{i}\;,$$

Equation (5) is the so-called logit model.

#### 3.2.2. Empirical Model and Variable Descriptions

We set up a binary logit model:

(6) $$W_{i}=\alpha_{0}+\alpha_{j}Score_{ij}+\sum_{s=1}^{4}\alpha_{s}H_{i}^{s}+\sum_{d=1}^{3}\alpha_{d}F_{i}^{d}+\varepsilon_{i},$$

where the binary explanatory variable *W_i_* represents the *i*th farmer’s willingness to adopt IPM technology (*W_i_* = 1 indicates willing to adopt; 0 otherwise); *Score_ij_*, as the key dependent variable in this study, represents the *i*th farmer’s score in the *j*th discipline test; $H_{i}^{s}$ is a vector of control variables, including gender, age, education years, and agrotechnical training experience; $F_{i}^{d}$ is a vector of family-related variables, including non-agricultural employment ratio, per capita arable land area, and average family income; *i* is the random error term; *α*_0_, *α_j_*, *α_s_*, and *α_d_* are parameters to be estimated. Table 2 summarizes the definitions and simple statistics of variables.

#### 3.2.3. Estimation Method

The dependent variable, the willingness to adopt IPM technologies, is a binary choice variable, which equals 1 or 0. In this case, we chose logit models for regression. Nevertheless, because of the linearity of the theoretical model and the binary values taken by the dependent variable, the equation represents a linear probability model (LPM). 

In this study, we first run a binary logit regression (Model ①) and estimate the marginal effects. Besides this, we re-estimate the data with a linear probability model (LPM) (Model ②) to check the robustness of the result. Considering regional differences in economics and agricultural production structure, we introduce regional dummy variables to control for regional heterogeneity and rerun the binary logit model (Model ③) and the LPM (Model ④). We also run separate binary logit models (models ⑤–⑧) on the scores for pest management, nutrient management, agro-environment, and cultivation technology.

## 4. Results

### 4.1. Descriptive Statistics

#### 4.1.1. Summary Statistics of Respondents

Table 3 summarizes the simple statistics of our respondents. Our respondents are rice producers aged from 28 to 79, with an average age of 54 and a median age of 55. More in detail, the majority, approximately 60%, of the respondents’ ages are above 50, indicating an aging trend of our respondents. Females count for 43.63% of the entire sample population. Approximately 75.84% of our respondents have received education for less than 6 years, which is lower than or equal to the elementary-school level. Only two respondents have received undergraduate education. Over 700 out of the 981 respondents have been producing rice for over 20 years, indicating sufficient experience in rice production. Besides this, 46.48% of the respondents have attended farm field school at least once. The mean planting area of rice per capita is 0.13 acres (about 2 Chinese mu), with a minimum of 0.01 and a maximum of 2.30. Approximately 96% of our respondents’ per capita rice planting area is smaller than 0.3 acres (about 5 Chinese mu). Overall, our respondents are relatively older, barely educated, experienced, and most full-time and small-scale rice producers, who display the typical characteristics of Chinese farmers.

#### 4.1.2. Agricultural Production Knowledge and Farmers’ Willingness to Adopt IPM Technology

Based on the scores of disciplines, we categorized the sample farmers to reveal the relationship between agricultural production knowledge and farmers’ willingness to adopt IPM technology in Table 4. Either from the perspective of the comprehensive test or each disciplinary test, farmers with higher scores are more willing to adopt IPM technology compared to the ones with lower scores. For instance, the proportion of farmers with comprehensive test scores higher than 180 points willing to adopt IPM technology is 17% higher than their counterparts with comprehensive test scores lower than 140 points. Besides this, 5% more farmers with a score higher than 50 points are willing to adopt IPM technology, compared to farmers with a score lower than 50 points in the discipline of nutrient management.

#### 4.1.3. Other Factors and Farmers’ Willingness to Adopt IPM Technology

When analyzing how farmers’ agricultural production knowledge affects their willingness to adopt IPM technology, we consider respondents’ demographic information and agricultural production experience, such as gender, age, education level, agrotechnical training experience, non-agricultural employment ratio in the family, per capita arable land area of the family, and average family income. We list the categorized factors and farmers’ respective willingness to adopt IPM technology in Table 5. Females are less willing to adopt IPM technology compared to males. The majority of farmers under 55 years old are willing to adopt IPM technology. The proportion of farmers over 55 years old willing to adopt IPM technology decreases as their age increases, indicating that elder farmers are more reluctant to adopt IPM technology. Farmers’ willingness to adopt IPM technology positively correlates with their education level. Beyond the demographic information, agrotechnical training experience, non-agricultural employment ratio in the family, and per capita arable land area of the family, as well as family average income, all positively correlate with farmers’ willingness to adopt IPM technology.

### 4.2. Regression Results

From the descriptive statistics, we may argue that agricultural production knowledge and other factors are related to farmers’ willingness to adopt IPM technology. To identify how agricultural production knowledge affects farmers’ willingness to adopt IPM technology, we set up regression models for further research.

#### 4.2.1. Comprehensive Agricultural Production Knowledge and Farmers’ IPM Adoption Intention 

The binary logit model is estimated using STATA16. Table 6 summarizes the estimation results. Regression results are highly consistent with previous descriptive analyses. Besides this, the estimation results from the binary logit models are similar to those from the LPMs, indicating robust regression results. Farmers’ comprehensive agricultural production knowledge positively affects their willingness to adopt IPM technology at the 5% level. We state that the more comprehensive production knowledge farmers master, the more willing they are to adopt IPM technology. Adopting IPM technology may cause a certain degree of risk (uncertainty) to agricultural production; thus, farmers with a lower level of comprehensive agricultural production knowledge tend to avoid risk by not adopting IPM technology. By contrast, farmers with better knowledge of comprehensive agricultural production may know the operations of IPM better and have an estimation of how much IPM could benefit their agricultural production, and thus are more willing to adopt IPM technology. The results of this study verify our hypothesis.

Looking at individual variables, we find that gender, age, and agrotechnical training experience significantly influence farmers’ willingness to adopt IPM technology. Males are more willing to adopt IPM technology compared with females. One possible reason is that females are more involved in taking care of the family, apart from participating in agricultural production, and thus have limited time to learn new technologies, such as IPM technology. Another possible reason is that males are more likely to take risks for higher gain than females [37], and thus are more willing to apply new technologies, which may bring higher profits, such as IPM. When not controlling for the regional difference, age negatively impacts the willingness to adopt IPM technology, indicating that older farmers are less willing to adopt IPM technology. However, when including the regional dummy variable, the effect of age becomes statistically insignificant. Farmers receiving agrotechnical training are more willing to adopt IPM technology at the 5% level. Receiving agrotechnical training may promote and popularize new ideas and technologies in agriculture to farmer participants, thus helping their in-depth understanding of IPM technology and reducing their uncertainty about the technology.

Looking at family variables, we find that the non-agricultural employment ratio significantly affects farmers’ willingness to adopt IPM technology at the 1% level. Farmers with a higher family non-agricultural employment ratio are more willing to adopt IPM technology. One possible explanation is that farmers evaluate the risks of adopting IPM technology. If the income of a household depends less on agricultural production, the family can afford a higher risk in agricultural production, and thus are more willing to adopt IPM technology.

#### 4.2.2. Disciplinary Knowledge and Farmers’ IPM Adoption Intention

To further reveal the impact of agricultural production knowledge on farmers’ willingness to adopt IPM technology, we re-ran the binary logit model using each discipline score as one explanatory variable. As indicated by Table 7, models ⑤ and ⑥ do not control the regional difference, while models ⑦ and ⑧ control the regional difference. The signs and significance levels are consistent between models ⑤/⑥ and ⑦/⑧, except for age. The score for the pest-management discipline is positive and significant at the 1% level, indicating that as farmers master more knowledge about pest management, they are more willing to adopt IPM technology. However, the scores for the nutrient management, agro-environment, and cultivation technology disciplines are all insignificant. One possible reason for the insignificance is that farmers’ awareness of IPM technology mainly focuses on pest management, not realizing the connection between IPM technology and the other three disciplines. Another possible reason for the insignificant parameters is that farmers are more concerned about pest management, as pests and diseases may lead to severe crop failure, thus causing a huge loss to farmers’ income, which greatly emphasizes the importance of understanding pest management for farmers. For the individual, family, and control variables, the estimated signs and significance levels are similar with those in previous models.

## 5. Conclusions

The traditional theory of farmers’ behavior has always been in the leading position in existing research on the adoption of agricultural technology. In this article, we refer to the basic variable model of individual behavior and evolutionary economics, considering the effect of psychological cognition on the behavior theory of farmers and re-exploring the problem of farmers’ adoption of new agricultural technology from the perspective of agricultural production knowledge. We chose farmers’ adoption of integrated pest management measures as the research object. As integrated pest management is a complex principle involving multiple categories, farmers’ knowledge of multiple disciplines might affect their adoption. According to Steiro et al. [38], farmers’ adoption of more complex principles might be lowered because of farmers’ lack of respective knowledge. Thus, we investigate in this study how farmers’ compounded and disciplinary knowledge influences their adoption of integrated pest-management measures.

Based on survey data containing 981 farmers from the Anhui province of China, we ran logit and LPM models to explore how agricultural production knowledge influences farmers’ willingness to adopt IPM technology. Since our main objective was to determine the role of agricultural production knowledge on rice producers’ IPM adoption intention, we focused on how the knowledge is defined. Unlike previous literature that uses farmers’ educational level or training experience to represent their knowledge, in this study we gave an exam to rice-producing respondents and obtained their exam scores to represent their agricultural production knowledge level. Considering the complexity of IPM, we included multiple disciplines in the exam, including pest management, nutrient management, agricultural environment, and rice cultivation. The analyses were run in two steps: first with the comprehensive score containing all four disciplines and second with disciplinary scores. The results show that the score of comprehensive agricultural production knowledge has a significantly positive impact on farmers’ willingness to adopt IPM technology, which proves our hypothesis raised in the section Conceptual Framework. Considering the specific discipline of agricultural production knowledge, the score for pest management has a significant positive impact on farmers’ willingness to adopt IPM technology, while the scores of the other three disciplines have no significant impact. In addition, some of the individual- and family-characteristic variables, such as gender and the family non-agricultural employment ratio, also affect farmers’ willingness to adopt IPM technology to varying degrees. Allahyari et al. [19] infer that respondents’ technical knowledge positively influences their adoption of IPM. Lacking an accurate definition of technical knowledge, we assume that it represents respondents’ level of knowledge of how IPM is applied, which is similar to the comprehensive knowledge in our study. In this case, our results are consistent with Allahyari et al. [19]. Gautam et al. [7] explore the impact of training farmers in IPM and conclude that training increases farmers’ knowledge of insect pests, thus promoting their intention to apply IPM. Our conclusion that farmers’ knowledge of pest management positively influences their IPM adoption intention is consistent with Gautam et al. [7]. Liu et al. [23] evaluate orange farmers’ understanding of chemical pesticide residuals and conclude that a higher cognition of chemical pesticide residuals would enhance farmers’ willingness to adopt IPM technologies. Our conclusion does not follow Liu et al. [23], as we indicate no significant effects of agro-environment knowledge on farmers’ IPM adoption intention. One possible reason is that, since rice is a common staple grain with a lower economic value compared with fruits such as oranges, environmental friendliness is not a highly rewarded credence attribute. As rice farmers do not obtain many economic benefits from being environmentally friendly, they are less likely to learn environmental knowledge (which is proved by the test results as shown in Table 1 and Table 2) or be affected by environmental knowledge.

Though various works in the literature have investigated farmers’ IPM adoption, few have focused on the impact of farmers’ knowledge. The role of knowledge in farmers’ adoption of new technologies is of no doubt. Besides this, farmers’ knowledge could be increased through targeted training. Thus, clarifying the current situation of rice farmers’ knowledge as well as identifying the relationship between farmers’ disciplinary knowledge and their IPM adoption would assist the central and local governments in promoting IPM technologies. Based on this conclusion, we suggest that to popularize agricultural IPM technology, the government first needs to strengthen farmers’ agricultural production knowledge, especially focusing on pest-management knowledge. As farmers’ cognitive level improves, their decision-making behavior may change accordingly. Secondly, the government should better promote IPM technology using various methods, in combination with farmers’ individual and family characteristics.

Identifying the relationship between rice farmers’ comprehensive and disciplinary knowledge is the first step. In future research, we will investigate why/why not disciplinary knowledge influences farmers’ IPM adoption. In this study, we focus only on theoretical/background knowledge. In future research, we will include more categories of knowledge, such as knowledge about the application of IPM. We are also interested in the weights of the knowledge of each discipline in farmers’ adoption of IPM. Additionally, we focus on intention in this study, which might diverge from farmers’ actual behavior. In future research, we will pay attention to farmers’ actual IPM adoption instead of their intention only.

## Figures and Tables

**Table 1:** Agricultural production knowledge research design and simple statistics.

	Comprehensive Test	Knowledge Disciplines			
		Pest Management	Nutrient Management	Agro-Environment	Cultivation Technology
Full Score	400	100	100	100	100
Score	161	40	44	22	56

**Table 2:** Definitions and simple statistics of variables (mean SD).

Variable Name	Definitions	Mean	Std Dev.
**Explanatory variable**			
*W_i_*	The *i*th farmer’s willingness to adopt IPM technology 1 = Yes, 0 = No	0.49	0.50
**Key explanatory variables**			
*Score_i_*_0_	score of comprehensive test	160.87	44.94
*Score_i_*_1_	score of the pest management discipline	39.52	10.95
*Score_i_*_2_	score of the nutrient management discipline	43.78	22.55
*Score_i_*_3_	score of the agro-environment discipline	21.84	20.37
*Score_i_*_4_	score of the cultivation technology discipline	55.73	13.75
**Individual variables**			
*Gender*	1 = Female, 0 = Male	0.44	0.50
*Age*	year	54.23	10.53
*Education*	education years	4.26	3.94
*Training*	agrotechnical training experience 1 = Yes, 0 = No	0.46	0.50
**Family variables**			
*NA employment*	Non-agricultural employment ratio (%)	26.81	33.35
*Land*	Per capita arable land area (acre)	0.13	0.11
*Income*	Average family income (¥1000)	36.20	42.27

**Table 3:** Summarized statistics for respondents’ demographic characteristics.

Characteristics	Min	Max	Category	Sample Size	Percentage (%)
*Age*	28	79	(20,30]	1	0.10
			(30,40]	69	7.03
			(40,50]	326	33.23
			(50,60]	247	25.18
			(60,80]	338	34.45
*Education Years*	0	16	[0,6)	419	42.71
			[6,9)	325	33.13
			[9,12)	207	21.10
			[12,16)	28	2.85
			[16,19)	2	0.20
*Training*	0	1	0	525	53.52
			1	456	46.48
*NA employment*	0	100	0	544	55.45
			(0,20]	32	3.26
			(20,40]	75	7.65
			(40,60]	111	11.31
			(60,80]	165	16.82
			(80,100]	54	5.50
*Land Size*	0.01	2.30	(0,0.3]	942	96.02
			(0.3,0.6]	33	3.36
			(0.6,0.9]	5	0.51
			(0.9,2.3]	1	0.10

**Table 4:** The relationship between scores and farmers’ willingness to adopt IPM technology.

	Sample Size	Willing to Adopt IPM (%)
Integrated score		
(0,140]	349	42
(140,180]	337	46
>180	295	59
Score of pest management discipline		
(0,35]	325	49
(35,45]	377	54
>45	279	57
Score of nutrient management discipline		
(0,50]	501	46
>50	480	51
Score of agro-environment discipline		
(0,25)	343	44
[25,50]	604	57
>50	34	76
Score of cultivation technology discipline		
(0,50]	507	49
(50,75]	312	45
>75	162	54

**Table 5:** The relationship between farmers’ demographic information/agricultural production experience and willingness to adopt IPM technology.

	Sample Size	Willing to Adopt IPM (%)
Gender		
Female	428	42
Male	553	54
Age		
≤45	248	57
(45,55]	253	56
(55,60]	192	47
>60	288	36
Education years		
0	419	40
(0,6]	325	51
>6	237	60
Agrotechnical training experience		
Yes	456	54
No	525	44
Non-agricultural employment ratio in the family (%)		
[0,35]	196	30
(35,50]	203	48
(50,75]	327	48
>75	255	64
Per capita arable land area of the family (acre)		
(0,0.08]	301	44
(0.08,0.14]	357	48
>0.14	323	54
Average family income (¥1000)		
(0,16]	328	44
(16,46]	391	47
>46	262	57

**Table 6:** Regression results of farmers’ IPM adoption intention (comprehensive agricultural production knowledge).

Variables	Model ①	Model ②	Model ③	Model ④
**Key explanatory variables**				
*Score_i_*_0_	0.004 ** (0.002) [0.001] **	0.001 ** (0.000)	0.004 ** (0.002) [0.001] **	0.001 ** (0.000)
**Individual variables**				
*Gender*	−0.479 *** (0.160) [−0.108] ***	−0.110 *** (0.036)	−0.305 * (0.165) [−0.065]	−0.066 * (0.036)
*Age*	−0.018 ** (0.008) [−0.004] **	−0.004 ** (0.002)	−0.007 (0.008) [−0.002]	−0.002 (0.002)
*Education*	0.025 (0.020) [0.006]	0.006 (0.005)	0.023 (0.021) [0.005]	0.005 (0.005)
*Training*	0.323 ** (0.137) [0.073] **	0.072 ** (0.031)	0.379 *** (0.145) [0.081] ***	0.081 ** (0.031)
**Family variables**				
*NA employment*	0.014 *** (0.003) [0.003] ***	0.003 *** (0.001)	0.009 *** (0.003) [0.002] ***	0.002 *** (0.001)
*Land*	0.398 (0.683) [0.089]	0.094 (0.154)	0.001 (0.525) [0.000]	0.002 (0.112)
*Income*	0.001 (0.002) [0.000]	0.000 (0.000)	−0.001 (0.002) [−0.000]	−0.000 (0.000)
**Regional dummies**			Controlled	Controlled
*Intercept*	−0.705 (0.644)	0.343 ** (0.145)	−1.363 ** (0.678)	0.196 (0.145)
Sample size	981			

Note: Models ① and ③ represent logit estimates, while models ② and ④ represent respective LPM estimates. In models ① and ②, the regional differences are not controlled, while models ③ and ④ control the regional differences. Numbers in the parentheses are standard errors. Numbers in brackets are marginal effects; *, **, and *** indicates *p* < 0.10, 0.05, and 0.01.

**Table 7:** Regression estimates of farmers’ IPM adoption intention (specific discipline).

Variables	Model ⑤	Model ⑥	Model ⑦	Model ⑧
**Key explanatory variables**				
*Pest-management knowledge*	0.027 *** (0.007) [0.006] ***	0.006 *** (0.002)	0.026 *** (0.008) [0.006] ***	0.006 *** (0.002)
*Nutrient-management knowledge*	0.000 (0.004) [0.000]	0.000 (0.001)	0.003 (0.004) [0.001]	0.001 (0.001)
*Agro-environment knowledge*	−0.001 (0.004) [0.000]	−0.000 (0.001)	−0.003 (0.004) [−0.001]	−0.001 (0.001)
*Cultivation-technology knowledge*	0.001 (0.005) [0.000]	0.000 (0.001)	−0.000 (0.005) [−0.000]	−0.000 (0.001)
**Individual variables**				
*Gender*	−0.487 *** (0.161) [−0.108] ***	−0.111 *** (0.036)	−0.311 * (0.165) [−0.066] *	−0.067 * (0.036)
*Age*	−0.017 ** (0.008) [−0.004] **	−0.004 ** (0.002)	−0.007 (0.008) [−0.001]	−0.001 (0.002)
*Education*	0.024 (0.021) [0.005]	0.005 (0.005)	0.020 (0.021) [0.004]	0.004 (0.005)
*Training*	0.262 (0.139) [0.058]	0.058 * (0.031)	0.329 ** (0.148) [0.069] **	0.070 ** (0.032)
**Family variables**				
*NA employment*	0.014 *** (0.003) [0.003] ***	0.003 *** (0.001)	0.008 *** (0.003) [0.002] ***	0.002 *** (0.001)
*Land*	0.354 (0.641) [0.079]	0.084 (0.147)	−0. 042 (0.524) [−0.009]	−0.006 (0.105)
*Income*	0.001 (0.002) [0.000]	0.000 (0.000)	−0.001 (0.002) [−0.000]	−0.000 (0.000)
**Regional dummies**			Controlled	Controlled
*Intercept*	−1.187 * (0.684)	0.239 (0.151)	−1.671 ** (0.710)	0.134 (0.149)
Sample size		981		

Note: Models ⑤ and ⑦ represent the logit estimates parameters’ marginal effects, while models ⑥ and ⑧ represent LPM estimates. Model ⑦ and Model ⑧ control the regional differences, while Model ⑤ and Model ⑥ do not. Numbers in the parentheses are standard errors. Numbers in brackets are marginal effects; *, **, and *** indicates *p* < 0.10, 0.05, and 0.01.

## Data Availability

Not applicable.

## Supplementary Materials

The following supporting information can be downloaded at: https://www.mdpi.com/article/10.3390/ijerph192114292/s1, Table S1: Questions on Medium Indica Rice Production Technology.
