# Supplementary material for: Knowledge and Farmers’ Adoption of Green Production Technologies: An Empirical Study on IPM Adoption Intention in Major Indica-Rice-Producing Areas in the Anhui Province of China
Source: Int J Environ Res Public Health. 2022 Nov 1;19(21):14292. doi: 10.3390/ijerph192114292 (PMC9657904; doi:10.3390/ijerph192114292)
Supplement: Supplementary file 1 [file ijerph-19-14292-s001.zip › ijerph-1958839-supplementary.pdf]

Table S1. Questions on Medium Indica Rice Production Technology

Multiple Choice		Options		Answer
Q1	What disease does Picture 1 indicate?	① Rice false smut; ② Leaf blast; ③ Rice blast at panicle neck; ④ Black streaked dwarf disease; ⑤ Sheath blight; ⑥ Rice planthopper; ⑦ Leaf roller; ⑧ Chilo suppressalis; ⑨ Other diseases _____; 999=don't know	**Picture 1**	
Q2	How is the pest as in Picture 2 spreaded?	① Grey planthopper; ② Borer (Chilo suppressalis and Chilo suppressalis); ③ Leaf roller; 999=don't know	**Picture 2**	
Q3	What disease does Picture 3 indicate?	① Rice false smut; ② Leaf blast; ③ Rice blast at panicle neck; ④ Black streaked dwarf disease; ⑤ Sheath blight; ⑥ Rice planthopper; ⑦ Leaf roller; ⑧ Chilo suppressalis; ⑨ Other diseases _____; 999=don't know	**Picture 3**	
Q4	What disease does Picture 4 indicate?	① Rice false smut; ② Leaf blast; ③ Rice blast at panicle neck; ④ Black streaked dwarf disease; ⑤ Sheath blight; ⑥ Rice planthopper; ⑦ Leaf roller; ⑧ Chilo suppressalis; ⑨ Other diseases _____; 999=don't know	**Picture 4**	
Q5	What disease does Picture 5 indicate?	① Rice false smut; ② Leaf blast; ③ Rice blast at panicle neck; ④ Black streaked dwarf disease; ⑤ Sheath blight; ⑥ Rice planthopper; ⑦ Leaf roller; ⑧ Chilo suppressalis; ⑨ Other diseases _____; 999=don't know	**Picture 5**	

Q6	What disease does Picture 6 indicate?	① Rice false smut; ② Leaf blast; ③ Rice blast at panicle neck; ④ Black streaked dwarf disease; ⑤ Sheath blight; ⑥ Rice planthopper; ⑦ Leaf roller; ⑧ Chilo suppressalis; ⑨ Other diseases _____; 999=don't know	**Picture 6**	
Q7	In which condition does the disease as indicated in Picture 7 more possibly outbreak?	① Drought; ② High temperature and sunny; ③ Low temperature, cloudy and rainy; 999=don't know	**Picture 7**	
Q8	When is the approriate time to first control the disease as indicated in Picture 7?	① 3-5 days before the break; ② 3-5 days after the break; ③ Full heading stage; 4=grouting period; 999=don't know		
Q9	What pest does Picture 8 indicate?	① Rice planthopper; ② Leaf roller; ③ Chilo suppressalis; ④ Others _________; 999=don't know	**Picture 8**	
Q10	What pest does Picture 9 indicate?	① Rice planthopper; ② Leaf roller; ③ Chilo suppressalis; ④ Others _________; 999=don't know	**Picture 9**	

**True or False**		① True; ② False, 999=don't know	**Answer**
Q11	When there are many moths in the rice field, rice farmers need to apply pesticide immediately.		
Q12	Spiders in rice fields are pests that harm the growth of rice.		
Q13	Pesticides and herbicides are the only solutions for diseases, pests, and weeds.		
Q14	Low toxic pesticides are less toxic, for humans, but with low effects on diseases and pests, compared		
Q15	The rice false smut does not deteriorate rice quality.		
Q16	If the Urea fertilizer has a pungent ammonis smell, it is an inferior fertilizer.		
Q17	Top dressing should be applied in the morning when there is dew.		
Q18	Exposing the field to the sun (pump the water out of the rice field) for at least ten days can reduce rice tillering and prevent lodging.		

Q19	After transplanting, the more tillers in the field, the better.		
Q20	Increasing the usage of potassium fertilizer would cause the rice lodging.		
**Multiple Choices**		**Options**	**Answer**
Q21	What's the correct seed-soaking method for hybrid rice?	①Soak day and night;<br>②Soak day only;999=dont' know	
Q22	What's the preferred density for planting conventional hybrid rice?	① 5.25 inches X 10.50 inches<br>② 7.87 inches X 10.50 inches;999=don't	
Q23	Which one is prefrred in rice tillering perid?	①More water;②Less water;③The same	
Q24	Do you think it is appropriate to expose the field to the sun for at least ten days during the late phase of the tillering period?	1=Yes;0=No;999=don't know	
Q25	If not, why？ ______________________________		
Q26	Have you counted the number of tillers in the rice field?	1=Yes;0=No;999=don't know	
Q27	What's the favorite average number of tillers at the late phase of tillering?	①Less than 10; ②10-15;<br>③More than 15; 999=don't know	
Q28	What's the approriate time to cut off water before harvest?	①10 days ahead of harvest;②20 days ahead of harvest;③30 days ahead of harvest;999=don't know	
Q29	Is it better to prevent diseases/pests before the outbreak or apply pesticides after the outbreak?	①Prevention before outbreak; ②Treatment after outbreak;999=don't know	
Q30	Pesticide safety interval refers to:	①Interval from harvest;②Iterval between two applications of pesticides;<br>999=don't know	
Q31	Which one of the production of harmless agricultural products, green agricultural products, and organic agricultural products prohibits the usage of pesticides?	①The harmless agricultural products;②The green agricultural products;③The organic agricultural products;999=don't know	

Q32	Which on of the pest treatment can be applied in the morning with dew?	① Rice leaf roller; ② Rice sheath blight; ③ Rice false smut; 999=don't know	
Q33	The first suitable period for the control of rice false smut is____	①7-10 days ahead of the rupturing stage; ②3-4 days ahead of the rupturing stage stage; ③The full heading stage; 999=don't know	
Q34	What should be avoided when using abamectin to control "rice leaf roller"?	①High temperature; ②Strong sunshine; ③Mix with other chemical pesticides; 999=unknown	
Q35	What does IPM refer to?	① Using chemical pesticides; ②Using chemical pesticides and biopesticide; ③ Using chemical pesticides, biopesticides, pest-proof lamp, and exposing the field to sunshine, etc; ④Other; 999=don't know	
Q36	What one of the following operations is better?	①Continuously use the same type of pesticide; ②Rotatingly use different types of pesticides; ③Indifferent; 999=don't know	
Q37	Do you wear face mask and long-sleeve clothes when applying chemical pesticides?	①Yes;②No;999=don't know	
Q38	Do you consider the wind direction when applying pesticides?	①Yes;②No;999=don't know	
Q39	Do you clean and wash the spraying machne after applying pesticides?	①Yes;②No;999=don't know	

Q40	How do you read the pesticide instructions?	① Apply the pesticided according to self experience, without reading the instructions; ② Just pay attention to the dose and dilution method;③Read the instruction carefully, and check other documents to confirm the aunthenticity of the pesticide;④Confirm the active ingredients and methods of use; ⑤ Ask the agricultural materials distributor for usage method, not reading the instructions;⑥ Other	
Q41	When there is a typhoon, what would happen to the rice planthopper?	①Increase;②Decrease;③Indifferent; 999=don't know	
Q42	Which one of the following fertilizers are the most volatile?	① Urea; ② Ammonium bicarbonate; ③ Ammonium chloride; ④ Superphosphate; 999=don't know	
Q43	Have you heard about the greenhouse gases?	①Yes;②No	
Q44	How does the greenhouse gas change with more usage of chemical fertilizers?	①Increase; ②Decrease; ③No change; 999=don't know	
Q45	Would the usage of chemical fertilizers pollute underground water and the rivers?	①Yes;②No;999=don't know	
**Fill in the blanks**		**Answer**	
Q46	What percentage do you think the output yield would reduce when chemical fertilizers are reduced by 50%?	______________________	
Q47	What percentage do you think the output yield would reduce with no chemical fertilizers at all?	______________________	
